# Myeloid cells are involved in tumor immunity, metastasis and metabolism in tumor microenvironment

**DOI:** 10.1007/s10565-025-10012-y

**Published:** 2025-03-25

**Authors:** Chenbo Zhang, Ying Song, Huanming Yang, Kui Wu

**Affiliations:** 1 College of Pharmaceutical Science, Zhejiang University of Technology, Hangzhou, 310000 China; 2 HIM-BGI Omics Center, Hangzhou Institute of Medicine (HIM), Chinese Academy of Sciences, Hangzhou, 310000 China; 3 Zhejiang Cancer Hospital, Hangzhou Institute of Medicine (HIM), Chinese Academy of Sciences, Hangzhou, 310022 Zhejiang China; 4 BGI Genomics, Harbin, 150023 Heilongjiang China; 5 Guangdong Provincial Key Laboratory of Human Disease Genomics, BGI Research, Shenzhen, 518083 China

**Keywords:** Tumor microenvironment, Myeloid cells, Cancer metastasis, Glucose metabolism, Amino acid metabolism

## Abstract

Bone marrow-derived cells in the tumor microenvironment, including macrophages, neutrophils, dendritic cells, myeloid-derived suppressor cells, eosinophils and basophils, participate in the generation, development, invasion and metastasis of tumors by producing different cytokines and interacting with other cell types, and play a pro-tumor or anti-tumor role in regulating tumor immunity. Due to the complexity of cell types in the tumor microenvironment and the unknown process of tumor development and metastasis, cancer treatment to achieve better survival status remains challenging. In this article, we summarize the effects of myeloid cells in tumor microenvironment on tumor immunity, cancer migration, and crosstalk with metabolism (including glucose metabolism, lipid metabolism, and amino acid metabolism), which will help to further study the tumor microenvironment and seek targeted therapeutic strategies for patients.

**Graphical abstract:**

Myeloid cells in the tumor microenvironment exhibit diverse biological functions, influencing tumor initiation and progression. Myeloid cells are involved in tumor immunity and metastasis processes, interacting with other immune cell types to affect the tumor immune response. The crosstalk between glucose metabolism, lipid metabolism, amino acid metabolism, and myeloid cells impacts tumor immunity and metastasis.

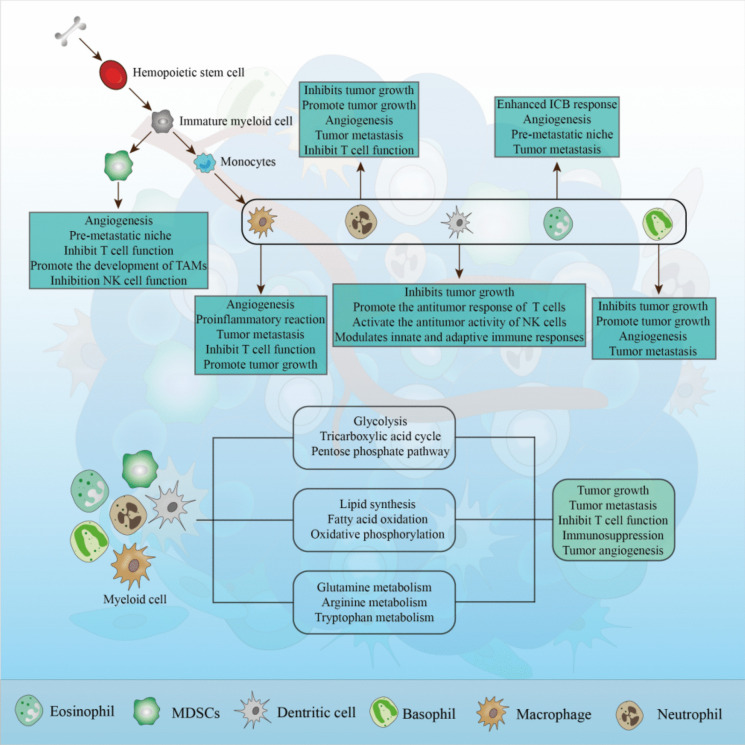

## Introduction

Tumor microenvironment (TME) is closely related to tumor development, metastasis and metabolism. In recent years, with the rapid development of single-cell and spatial transcriptomics technologies, the cellular structure and components of tumor microenvironment have been dissected, extends our understanding that the interaction between the TME and cancer cells and non-cancer cells determines the occurrence, development, metabolism, metastasis and clinical therapeutic process of tumors.

**Fig. 1:**
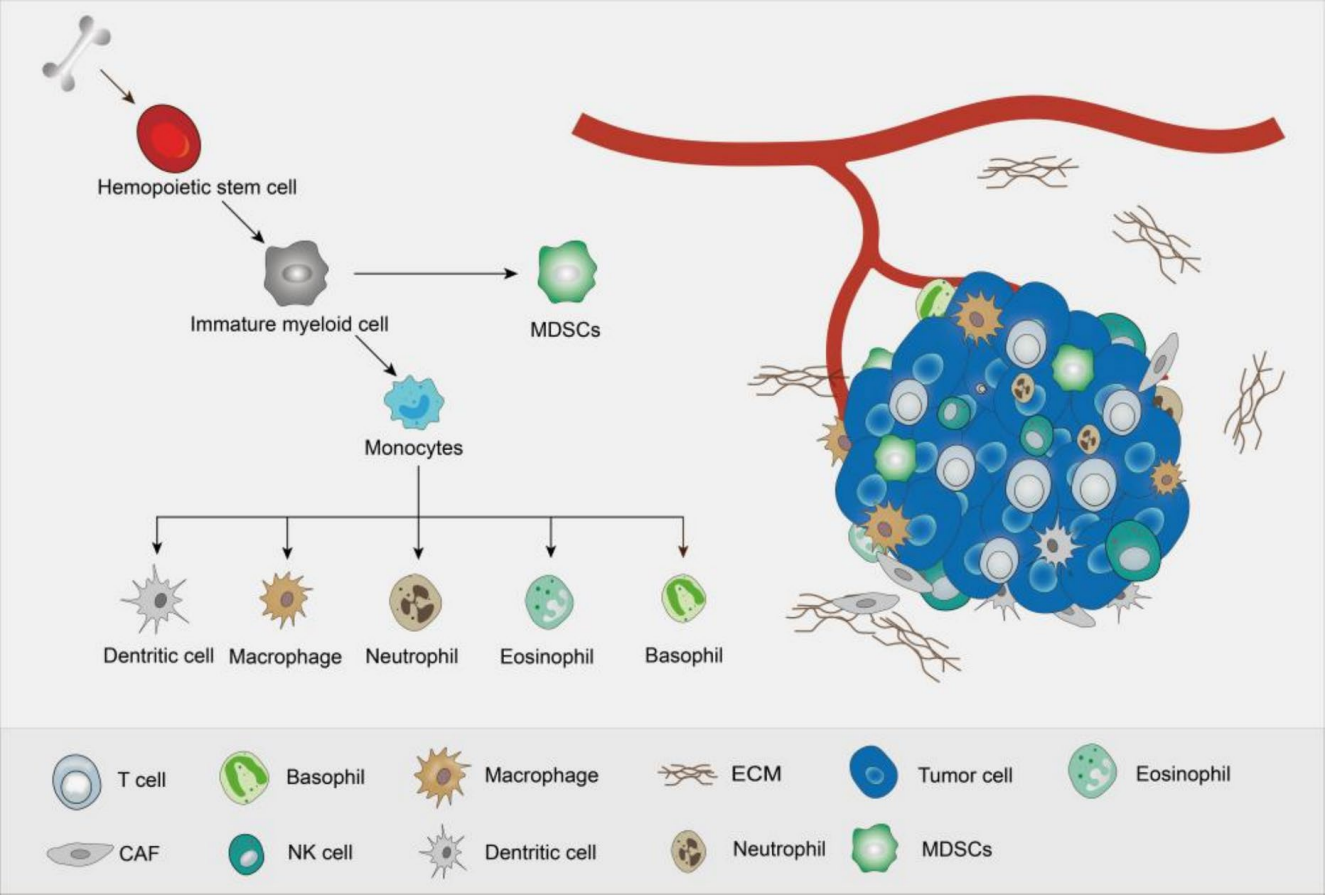
Hematopoietic stem cells derived from bone marrow differentiate into immature myeloid cells, normally, immature myeloid cells differentiate into monocytes, and subsequently into macrophages, granulocytes and dendritic cells, however, under the influence of certain diseases such as infection, inflammation, and tumors, immature myeloid cells will differentiate into myeloid-derived suppressor cells with immunosuppressive functions. These cells, together with tumor cells, immune cells, extracellular matrix and various cytokines, constitute the tumor microenvironment

TME refers to the cellular environment in which tumors are located, which is composed of complex and different cellular components, including immune cells, lymphocytes, fibroblasts, extracellular matrix, blood vessels, cells derived from bone marrow, and various cell signaling factors (Fig. 1). These cells and signaling factors can not only inhibit tumor growth and metastasis, but also promote tumor growth and immune evasion. Due to the different underlying mechanisms of various cells, their role in tumor development and cancer suppression is still a major topic in the research community (Arneth 2019; Vitale et al. 2019; Xiao and Yu 2021).

Among the studies on various cell types in the TME, bone marrow-derived cells including macrophages, neutrophils, dendritic cells, myeloid-derived suppressor cells, and eosinophils and basophils (de Visser and Joyce 2023) are also essential factors affecting TME. Therefore, this review aims to reveal the interconnections between myeloid cells and tumor immunity, metastasis, and metabolism to provide a comprehensive insight of tumoral society.

## Modulate tumor immunity

### Tumor-associated macrophages (TAMs)

Macrophages are part of the innate immune regulation and can phagocytose pathogens and digest cell debris. They can also activate other types of immune cells to resist the invasion of pathogens (Wang et al. 2022a). TAMs are an important component of the tumor microenvironment and participate in processes such as tumor progression, metastasis, and angiogenesis (Mills et al. 2016; Yin et al. 2019; Zhou et al. 2020b). Because TAMs are highly plastic, they can further polarize into classically activated macrophages (M1) and selectively activated macrophages (M2). M1 macrophages, also known as pro-inflammatory macrophages, are primarily activated by lipopolysaccharides (LPS), activated Toll-like receptors (TLRs), and interferon-gamma (IFN-γ) secreted by Th1 cells, cytotoxic T lymphocytes (CTLs), and natural killer (NK) cells, with strong antigen presentation and phagocytosis. And M1 macrophages can produce tumor necrosis factor-alpha (TNF-α) and pro-inflammatory cytokines such as IL-6, IL-12, and IL-18, express high levels of Major Histocompatibility Complex Class II (MHC II) and CD68, and inhibit tumor cell growth and proliferation. Additionally, M1 macrophages stimulate T cell activity to induce anti-tumor immune responses, which is why they are considered anti-tumor macrophages. However, under the influence of cytokines such as IL-4, IL-10, IL-13, transforming growth factor-beta (TGF-β), and macrophage colony-stimulating factor (M-CSF), TAMs polarize into M2 macrophages, which promote tumor development. M2 macrophages have high expression of mannose receptors and low levels of MHC II, and they release immunosuppressive factors such as IL-10, TGF-β, and arginase-1 (Arg-1). They exhibit poor antigen-presenting capacity, promote tumor cell growth, and have immunosuppressive effects (Li et al. 2021a; Wang et al. 2024; Zhang et al. 2023). For example, in liver cancer, overexpression of retinoic acid-inducible gene I (RIG-I) can promote M2 polarization of peritoneal macrophages in mice through the RIG-I/MAVS/TRAF1/NF-κB pathway, thus inducing apoptosis of liver cancer cells (Zhou et al. 2020a).

Although substantial evidence suggests that TAMs promote cancer progression, their role varies significantly across different tumor types due to the presence of different subtypes. For example, in lung cancer, a meta-analysis showed that lung cancer patients with increased tumor-infiltrating M1 macrophage density had better 3-year and 5-year overall survival, while patients with increased tumor-infiltrating M2 macrophage density had poorer overall survival (Wu et al. 2016). In colorectal cancer, IL-10 promotes tumor growth and metastasis by inducing M2 polarization of TAMs (Liu et al. 2020), indicating that the M2 subtype is associated with poor prognosis in colorectal cancer. Given the plasticity of TAMs, M2 macrophages can be re-polarized into M1 macrophages. For example, inhibiting the Hedgehog signaling pathway in M2 macrophages can reduce the abundance of M2 macrophages and promote reprogramming of M2 to M1 macrophages, thereby decreasing the immunosuppressive effects of M2 macrophages (Hinshaw et al. 2021). Other studies have shown that the tumor suppressor gene SHISA3 can promote M1 polarization of macrophages and act on tumor cells to limit tumor growth (Zhang et al. 2024). Therefore, re-polarizing M2 macrophages into M1 macrophages or directly polarizing macrophages into M1 may serve as a novel strategy for tumor immunotherapy.

In addition, TAMs contribute to tumor development by promoting blood vessel formation and reacting with other types of immune cells. In non-small cell lung cancer and melanoma, TAMs are closely related to angiogenesis. The increase of M2 macrophages can promote the expression of vascular endothelial growth factor A (VEGF-A) and vascular endothelial growth factor C (VEGF-C) in non-small cell lung cancer cells, Moreover, TAM-derived adrenomedullin induces the phosphorylation of endothelial nitric oxide (NO) synthase in endothelial cells and polarizes macrophages to the M2 phenotype in an autocrine manner, promoting angiogenesis and melanoma growth (Chen et al. 2011; Hwang et al. 2020). M2 macrophages can secrete immunosuppressive molecules, such as IL-10, TGF-β, and human leukocyte antigen G, into the TME (Komohara et al. 2016). They can also directly interact with myeloid suppressor cells (MDSC) and inhibit T-cell-mediated anti-tumor response (Boutilier and Elsawa 2021). Moreover, TAMs mediate tumor immunosuppression when cellular immune checkpoint ligands are expressed, directly inhibiting T cell function. They also secrete chemokines such as CCL2, CCL3, CCL4, CCL5 and CCL20, which contribute to the recruitment of Tregs in the tumor microenvironment (Cassetta and Pollard 2020). In addition, TGF-β affects anti-PD-1/PD-L1 immunotherapy by inhibiting the expression of PD-L1 and T cell activation (Batlle and Massagué 2019). However, TGF-β derived from TAMs inhibits T cell activity through the phosphorylation of Smad2/3 proteins and the suppression of mitochondrial respiration (Dimeloe et al. 2019), thereby affecting T cell immune function.

### Tumor-associated neutrophils (TANs)

Neutrophils are innate immune cells derived from bone marrow and are an important line of defense against infection and maintaining homeostasis. They are also the most abundant immune cells in the circulating system (Quail et al. 2022). Neutrophils infiltrated in tumors are involved in tumor initiation, metastasis and immunosuppression with their phenotypes and functions (Que et al. 2022). Studies have shown that TANs can prevent the development of cancer by directly killing tumor cells or activating innate and adaptive immunity (Jaillon et al. 2020; Que et al. 2022). For example, it can inhibit tumor cell proliferation through the Fas ligand/Fas pathway (Sun et al. 2018), it can also activate innate immunity and adaptive immunity to exert anti-tumor effects. In the early stages of head and neck cancer progression, TANs metastasize into lymph nodes to initiate anti-tumor responses by stimulating T cells (Pylaeva et al. 2022). Neutrophils can also activate dendritic cells and T cells by virtue of their ability to present antigens or by releasing neutrophil extracellular traps (NETs) (Berger-Achituv et al. 2013; Sionov et al. 2015; Tillack et al. 2012). In addition, neutrophils can also express a variety of signaling factors that promote tumor growth and development, including epidermal growth factor (EGF), hepatocyte growth factor (HGF) and platelet-derived growth factor (PDGF) (Jaillon et al. 2020) to promote tumor growth and development and promote tumor angiogenesis. Studies have shown that co-culture TANs with TAMs can produce higher levels of oncostatin M and IL-11, both of which activate STAT3 signaling in intrahepatic cholangiocarcinoma (ICC); Thereby enhancing the proliferation and invasion ability of ICC cells in vitro and tumor progression in the ICC mouse xenograft model (Zhou et al. 2021). In an NK cell-depleted mouse model, neutrophils transform into a tumor-promoting phenotype, thereby upregulating the expression of VEGF-A and promoting tumor growth and angiogenesis (Ogura et al. 2018).

### Dendritic cells (DCs)

DCs is the main antigen presenting cell (APC) in the human body, which can recognize danger signals in inflammation and tumors, and present these signals to T lymphocytes to mobilize innate and adaptive immunity (Balan et al. 2018; Lu et al. 2022b), thereby controlling tumor growth and development. Human DC contains three main subpopulations: Plasmacytoid dendritic cells (pDCs), bone marrow-like dendritic cells CD141 and CD1c, also known as classical DCs (cDC1s, cDC2s). The pDCs subgroup may be in the immature stage, which triggers T cell response immediately after receiving the virus signal. cDC1s and cDC2s subsets stimulate T cells by secreting cytokines when activated in vitro (Balan et al. 2018; Ziegler-Heitbrock et al. 2010). DCs are considered an important component of the TME and can promote T cell anti-tumor responses. Lei et al. 2023. determined the transcriptomic signature of CD4 + T cell DC licensing in human cancers through scRNA-seq and flow cytometry, cognate interactions of CD4 + T cells and CD8 + T cells with cDC1s can promote CTLs responses and Th1 differentiation, thereby translating into T cell-mediated tumor suppression in multiple human solid tumor types. Some studies have also shown that cDC1s can transport tumor antigens into tumor drainage lymphatic vessels to activate T cells, and produce chemokines to recruit T cells into the TME, at the same time, IL-12 is secreted to stimulate the ability of tumor-infiltrating T cells (Böttcher et al. 2018). However, the interaction between cDC1s and NK cells in the TME may lead to their mutual activation and promote anti-tumor immunity (Böttcher et al. 2018). For example, IL-12 secreted from DCs can enable NK cells to produce IFN-γ and activate the anti-tumor activity of NK cells (Mittal et al. 2017), while NK cells can deliver the IFN-γ produced to DCs 24 h after infection to maintain the production of IL-12 (Alexandre et al. 2016).

### Myeloid-derived suppressor cells (MDSCs)

MDSCs are derived from a heterogeneous population of immature myeloid cells generated from hematopoietic stem cells (HSCs) in the bone marrow (Li et al. 2021c; Solito et al. 2011) According to different surface markers, MDSCs are mainly divided into two subtypes: granulocytic MDSC (G-MDSC), also known as polymorphonuclear MDSC (PMN-MDSC) and mononuclear MDSC (M-MDSC) (Chen et al. 2022; Talmadge and Gabrilovich 2013; Yang et al. 2020b; Zhang et al. 2019). In TME, a variety of factors such as granulocyte colony-stimulating factor (G-CSF), M-CSF, granulocyte macrophage colony-stimulating factor (GM-CSF), LPS, IL-6, IFN-γ, VEGF and prostaglandin E2 (PGE2) induce the differentiation of MDSCs (Zhao et al. 2016). However, MDSCs have strong immunosuppressive functions and are associated with poor clinical outcomes (Veglia et al. 2021). MDSCs perform immunosuppression through interactions with T cells, macrophages, NK cells, regulatory T cells, and DC cells (Pramanik and Bhattacharyya 2022). For example, studies have shown that M-MDSCs affect the activation of early CD8 T cells by reducing T cell proliferation, enhancing IFN-γ production and reducing IL-2 responsiveness, and its derived NO impairs T cell extravasation and tissue infiltration by downregulating CD44 and CD162 on T cells (Schouppe et al. 2013). In intestinal cancer, doublecortin-like kinase 1 promotes immunosuppression in the TME by recruiting MDSCs (Yan et al. 2023). Furthermore, IL-10 produced by MDSCs can differentiate helper T cells into a Th2 phenotype and produce high levels of IL-4, thus promoting the development of TAMs (DeNardo et al. 2009; Zhao et al. 2016). In addition to suppressing immune cells, MDSCs can also support tumor angiogenesis, promote epithelial-to-mesenchymal transition (EMT) (Li et al. 2021c). For example, high motility group box protein 1 (HMGB1) secreted by MDSCs promotes the migration of human lung microvascular endothelial cells and the formation of capillary-like tubes through the ERK/P38/Src signaling pathway (Liu et al. 2017; Wang et al. 2019). Moreover, studies have shown that MDSCs can secrete matrix metalloprotein (MMP), and MMP-9 can increase the bioavailability of VEGF and promote the growth and maintenance of tumor blood vessels (Jacob and Prekeris 2015; Lugano et al. 2020).

### Eosinophils and basophils

Eosinophils are BM-derived granulocytes with multifunction and high heterogeneity. Eosinophils are the main component of type II immune responses and can secrete many factors, such as cytotoxic granules, lipids, growth factors, cytokines, and chemokines (Jacenik et al. 2023). Previous research on eosinophils mainly focused on allergic diseases and parasitic infections. However, in recent years, more and more studies have shown that eosinophils can interact with different immune cells in the TME and are components of the immune landscape of various tumor types (Arnold et al. 2020; Grisaru-Tal et al. 2021, 2020; Kienzl et al. 2020). For example, eosinophils can promote the recruitment of CD4 + and CD8 + T cells into the TME, and the GM-CSF-IRF5 signaling axis in eosinophils can also activate T cell responses to promote anti-tumor immunity (Arnold et al. 2020; Grisaru-Tal et al. 2021). Studies have also shown that in patients with triple-negative breast cancer, eosinophils promote the immune checkpoint blockade (ICB) response by activating CD8 + T cells; at the same time, the cooperation of eosinophils and IL-5-producing CD4 + T cells can also enhance the ICB response (Blomberg et al. 2023).

Basophils develop from HSCs in the bone marrow and can produce several angiogenic factors such as VEGF, angiopoietin and cysteine leukotriene C4, In addition, human basophils can also express IL-4, IL-13, IFN-γ cytokines, and receptors for IL-3, IL-33, GM-CSF (Marone et al. 2020). Since basophils can secrete different cytokines, they may have pro-tumor and anti-tumor effects. For example, basophils migrate to lymph nodes and secrete IL-4, promoting the differentiation of naive CD4 + T cells into a Th2 phenotype, and can also interact as APC and T cells to drive Th2 cell differentiation (Marone et al. 2020). Studies have also shown that intratumoral basophils enhance the infiltration of T cells by producing chemokines CCL3 and CCL8, confirming the important role of basophils in tumor rejection (Sektioglu et al. 2017). Furthermore, mouse basophils can secrete TNF-α and histamine to exert anti-tumor effects, and the granzyme B released by basophils has a cytotoxic effect on cancer cells (Marone et al. 2020).

## Effect on tumor metastasis

### TAMs and tumor metastasis

**Fig. 2:**
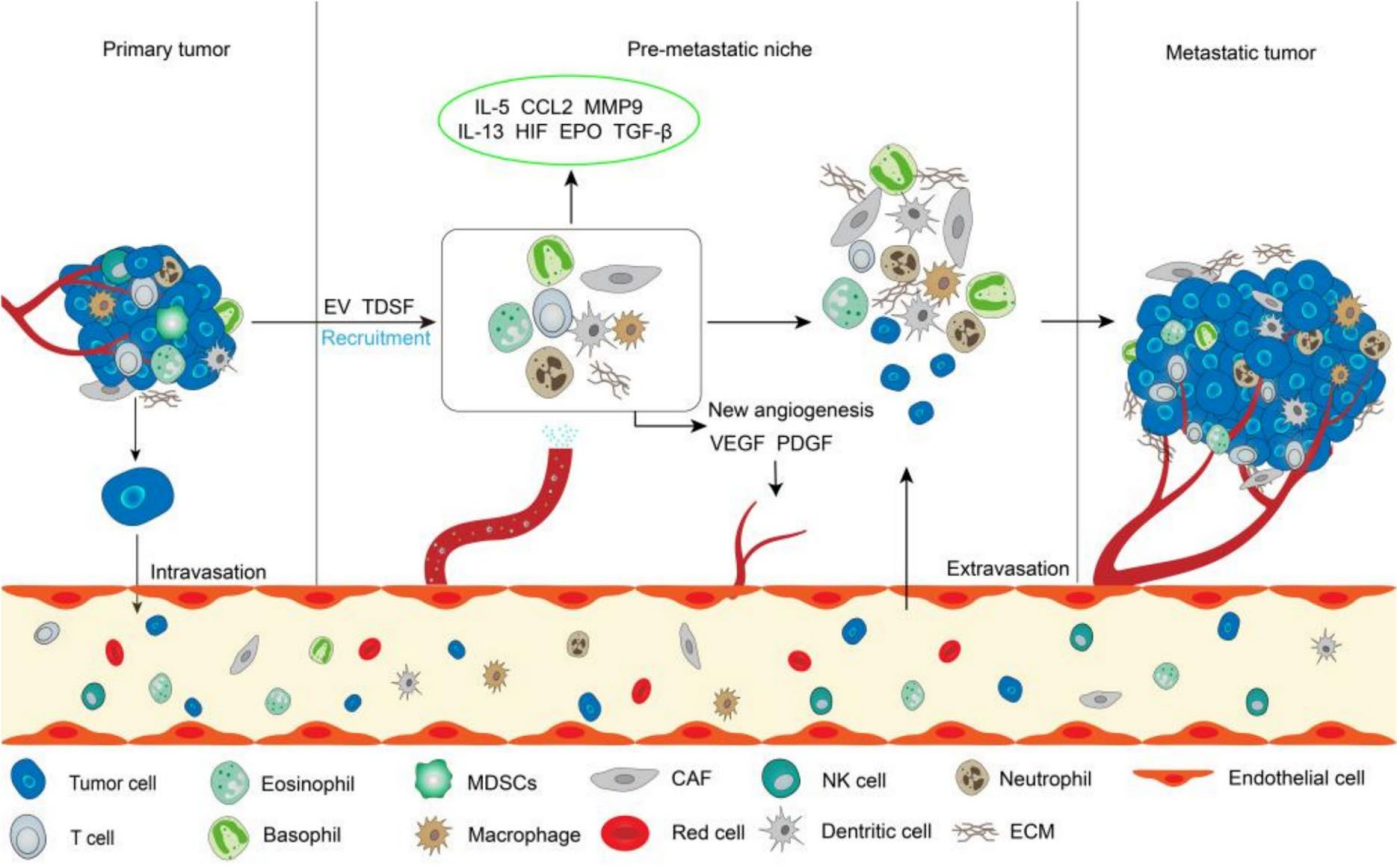
Primary tumor cells intrainfiltrate into the vasculature under the intervention of certain signaling factors, and then invade surrounding or distant tissues under the influence of blood flow. The process of metastasis is roughly divided into intravasation, formation of pre-metastatic niche, angiogenesis, epithelial-mesenchymal transition, extravasation, and ultimately leads to tumor metastasis. Tumor-derived extracellular vesicles (EVs) and tumor-derived secreted factors (TDSF) can recruit myeloid cells and release different types of cytokines such as IL-5, CCL2, EPO, TGF-β and angiogenic factors VGEF, PDGF, promoting premetastatic niche formation

Metastasis is the process in which tumor cells escape immune surveillance from the primary site of the tumor and spread to other sites through blood circulation or lymphatic metastasis. Metastasis includes processes such as the formation of the premetastatic niche (PMN), angiogenesis, EMT, invasion, intravasation, extravasation, metabolic reprogramming, and immune regulation (Fig. 2) (Castaneda et al. 2022; de Visser and Joyce 2023). EMT refers to the ability of cancer cells to lose their epithelial characteristics to acquire a mesenchymal phenotype with the ability to invade and migrate. After undergoing the EMT process, tumor cells acquire enhanced migration and invasion abilities, thereby promoting tumor metastasis. Studies have shown that cancer cells after EMT can secrete GM-CSF to activate macrophages to the TAMs phenotype, while activated macrophages induce EMT of cancer cells by secreting CCL18, this positive feedback loop is associated with metastasis of breast cancer cells (Su et al. 2014). TAMs induce EMT to enhance the invasion of colorectal cancer by regulating the JAK2/STAT3/miR-506-3p/FoxQ1 axis (Wei et al. 2019). In contrast, cytoplasmic polyadenylation element binding protein 3 (CPEB3) disrupts the crosstalk between colorectal cancer cells and TAMs by targeting the IL-6R/STAT3 signaling pathway, thereby inhibiting EMT (Zhong et al. 2020). In lung adenocarcinoma, IL-6 secreted by TAMs activates the JAK2/STAT3 pathway, and STAT3 can activate the expression of C/EBPβ, thereby enhancing the expression of IL-6. This forms a positive feedback loop of IL6-STAT3-C/EBPβ-IL6 in TAMs. Additionally, IL-6 promotes lung cancer progression and metastasis both in vivo and in vitro by activating the EMT pathway (Hu et al. 2024).

The formation of new blood vessels plays a crucial role in tumor metastasis. Cancer cells spread through the tumor vasculature, leading to tumor metastasis. TAMs directly or indirectly affect tumor vasculature and participate in angiogenesis by secreting pro-angiogenic factors and inflammatory factors, including VEGF-A, MMP, EGF, TGF-β, and TNF-α (Lu et al. 2024). For example, the infiltration of TAMs in the tumor microenvironment leads to the increase in CXCL8, which in turn promotes bladder cancer cells to secrete MMP-9, VEGF, and E-cadherin, enhancing the migration and angiogenic capacity of bladder cancer cells (Wu et al. 2020). In pancreatic ductal adenocarcinoma, exosomes derived from M2 macrophages can promote angiogenesis in mouse aortic endothelial cells in vitro, increasing the vascular density in mice (Yang et al. 2021). In addition, the interaction between TAMs and the tumor vasculature, the tumor hypoxic environment, and the influence of perivascular cells on the phenotype of TAMs also promote tumor angiogenesis (Chu et al. 2024; Huang et al. 2024). Therefore, based on the relationship between TAMs and angiogenesis, targeted therapeutic strategies can be developed to inhibit tumor angiogenesis by suppressing the pro-angiogenic factors released by TAMs and the associated signaling pathways. Moreover, macrophages are one of the key factors in the formation of PMN. The formation of PMN is the microenvironment in which cells invade sites far away from the primary tumor (such as liver, lung, brain, bone marrow, etc.). Before the arrival of tumor cells, the molecular biological characteristics of these sites have been changed, making them more suitable for the growth of tumor cells (Peinado et al. 2017). For example, gastric cancer-derived LPS-binding protein mediates crosstalk between primary gastric cancer cells and the intrahepatic microenvironment by promoting the secretion of TGF-β1 in intrahepatic macrophages, thereby inducing the formation of intrahepatic fibrotic PMN to promote liver metastasis of gastric cancer (Xie et al. 2023). Recent studies have also found that circ-0034880-rich extracellular vesicles can promote the interaction between primary tumor cells and tumor-promoting macrophages, thereby promoting PMN formation and colorectal cancer liver metastasis (Zhou et al. 2024).

### Other myeloid cells and tumor metastasis

In the early stages of metastasis, neutrophils provide a way for cancer cells to escape by promoting angiogenesis (Jaillon et al. 2020), then they guide cancer cells into the blood circulation. In this process, neutrophil-derived cathepsin G activates insulin-like growth factor-1 to promote the entry of cancer cells into the bloodstream (Morimoto-Kamata and Yui 2017), while NETs can capture circulating cancer cells and promote the colonization of cancer cells in distant organs (Najmeh et al. 2017). NETs are extracellular mesh-like fibrous structures formed by neutrophils that support tumor progression and metastasis (Deng et al. 2021b; Park et al. 2016; Tohme et al. 2016). For example, Discoid Domain Receptor-1 on pancreatic ductal adenocarcinoma cells can induce the recruitment of TANs and the formation of NETs, thereby promoting cancer cell invasion and metastasis (Deng et al. 2021b). pDCs appear to be involved in lymph node metastasis of cancer cells. Studies have shown that the secretions of pDCs isolated from the TME of breast cancer may stimulate the expression of CXCR4, thereby promoting lymph node metastasis (Gadalla et al. 2019). Under pathological conditions such as inflammation or tumors, pDCs can be recruited from the blood to peripheral tissues (Monti et al. 2020), and migrate to lymph nodes and tissue damage sites by expressing chemical protein receptors (Vermi et al. 2005).

MDSCs have been shown to promote the metastasis of cancer cells through their immunosuppressive activity and promoting angiogenesis (Cole et al. 2023; Gabrilovich et al. 2012; Oh et al. 2013; Talmadge and Gabrilovich 2013), and can also promote immune escape of cancer by inhibiting effector T cell and Tregs activation and inhibiting NK cells (Gabrilovich et al. 2012). In colorectal cancer, VGEF-A secreted by colorectal cancer cells can stimulate TAMs to produce chemokine (C-X-C motif) ligand 1 (CXCL1) in the primary tumor, and CXCL1 recruits chemokine (C-X-C motif) receptor-2 (CXCR2)-positive MDSCs into liver tissue to form a pre-metastatic niche and promote liver metastasis of colon cancer (Wang et al. 2017a). However, the chemokine CCL2 secreted by CAFs recruits MDSCs to the tumor site in a CCR2-dependent manner to form a pre-metastatic niche, thereby promoting cancer metastasis (Wang et al. 2019; Yang et al. 2016). The eosinophile-related factor IL-5 can mediate the release of mature eosinophils into the blood and migration to related tissues (Diny et al. 2017). Zaynagetdinov R et al. found that the pro-metastasis effect of IL-5 in colon cancer and melanoma is related to the presence of pulmonary eosinophils, proving that pulmonary eosinophils provide a favorable pulmonary microenvironment for tumor cells in the early stage of metastasis (Zaynagetdinov et al. 2015). Eosinophilic peroxidase (EPO) released by eosinophils can induce fibroblasts and collagen deposition within the primary tumor, and promote the growth and metastasis of the primary tumor by reshaping the composition and function of ECM in TME (Panagopoulos et al. 2017). Furthermore, tumor cells overexpress the chemokine CCL22 by inducing eosinophils, and CCL22 can be used to recruit Tregs (Mailloux et al. 2010), helping tumors establish a pre-metastatic niche, thereby promoting tumor metastasis (Zaynagetdinov et al. 2015). Only a few studies have addressed the relationship between basophils and cancer metastasis. Basophils secrete CXCL8, the most abundant cytokine in tumor tissue, and have been shown to be critical for metastasis (Mishra et al. 2021). For example, early studies showed that knocking down CXCL-8 receptors CXCR1 and CXCR2 inhibited angiogenesis and invasion as well as melanoma metastasis and invasion (Singh et al. 2010). In the microenvironment of colon cancer, CXCL8 and its receptor CXCR2 can promote the development and metastasis of colon cancer cells (Lee et al. 2012). We summarize the effects of cytokines and chemokines produced by different cell types in myeloid cells on tumor formation, development, metastasis, and invasion (Table 1).

**Table 1:** Cytokines and chemokines produced by different cell types in myeloid cells influence tumor formation, development, metastasis and invasion

Cell type	Release of cytokines	Effects on TME	Source
TAMs	IL-6, IL-12, IL-18, TNF-α	Produces pro-inflammatory response	(Li et al. 2021a; Wang et al. 2024; Zhang et al. 2023)
	VEGF, CXCL8, CXCL12	Promote tumor growth	(Boutilier and Elsawa 2021)
	EGF	Promote tumor invasion	(Boutilier and Elsawa 2021)
	CCL2, CCL3, CCL4, CCL5, CCL20	Contributes to the recruitment of Tegs in the TME	(Boutilier and Elsawa 2021)
	TGF-β	Suppress T cell activity	(Fu et al. 2015)
	CCL18, IL-8	Induction of EMT in cancer cells	(Fu et al. 2015; Tohme et al. 2016)
	IL-1β	Activates tumor cells, promotes glycolysis process and accelerates tumor growth	(Lu et al. 2020)
TANs	IL-17	Recruit and activate neutrophils to kill cancer cells	(Chen et al. 2017)
	EGF, HGF, PDGF	Promote tumor growth, development and tumor angiogenesis	(Balan et al. 2018)
	PD-L1	Suppress immune function of T cells	(Wang et al. 2017b)
	NET, NET-DNA	Promote cancer growth and metastasis	(Deng et al. 2021b; Park et al. 2016; Tohme et al. 2016; Yang et al. 2020a)
	IL-17a, IL-2a	Enhance the migration, invasion and EMT of gastric cancer cells	(Li et al. 2019)
	Oncostatin M, IL-11	Improve ICC cell proliferation and invasion in vitro	(Zhou et al. 2021)
DCs	IL-12	Activates anti-tumor activity of NK cells	(Böttcher et al. 2018; Mittal et al. 2017)
MDSCs	IL-10	Promote the development of TAMs	(Schouppe et al. 2013; Zhao et al. 2016)
	NO	Diminished response to antibody treatment	(Stiff et al. 2018)
	HMGB1	Promote human lung microvascular endothelial cell migration and capillary-like tube formation	(Liu et al. 2017; Wang et al. 2019)
	MMP-9	Promote the growth and maintenance of tumor blood vessels	(Jacob and Prekeris 2015; Lugano et al. 2020)
	CCL2, IL-1β, CXCL8, CXCL2, GM-CSF, ANGPT1, ANGPT2	Produce a pro-angiogenic environment within tumors	(Chun et al. 2015; Lugano et al. 2020; Obermajer et al. 2011)
	TGF-β	Promote cancer metastasis	(Wang et al. 2019)

Cell type	Release of cytokines	Effects on TME	Source
Eosinophil	IL-5	Promote cancer metastasis	(Zaynagetdinov et al. 2015)
	EPO	Promote primary tumor growth and metastasis	(Panagopoulos et al. 2017)
	CCL6	Recruit tumor cells and promote tumor metastasis	(Li et al. 2021b; Zhu et al. 2018)
	CCL22	Recruit Tregs and help tumors establish a PMN	(Mailloux et al. 2010; Panagopoulos et al. 2017)
	VEGF, IL-3, IL-8	Angiogenic factors.Promote angiogenesis in the TME	(Lee et al. 2023)
Basophil	IL-4	Promote the differentiation of naive CD4 + T cells into Th2 phenotype; promote bone metastasis of breast cancer	(Marone et al. 2020)
	CCL3, CCL8	Enhance T cell infiltration	(Castaneda et al. 2022)
	TNF-α, Histamine	Anti-tumor effect	(Marone et al. 2020)
	Granzyme B	Cytotoxic effect on cancer cells	(Marone et al. 2020)
	CXCL8	Promote the development and metastasis of colon cancer cells	(Singh et al. 2010)
	IL-13	Promote cancer metastasis; participate in the activation of M2 macrophages	(Barderas et al. 2012; Fujisawa et al. 2009; Mantovani et al. 2005)

## Regulating metabolism in the tumor microenvironment

### Glucose metabolism

TAMs are the main cell type that consumes glucose in the TME. Since macrophages have different subtypes, their glucose metabolism characteristics are also different. M1-polarized macrophages primarily rely on glucose and its conversion to lactate, the production of reactive oxygen species (ROS), and the generation of NO to carry out tumor killing. This process occurs under the stimulation of the cytokines IFN-γ, TNF, and LPS, and involves the intracellular shift toward aerobic glycolysis, ROS generation, disruption of the tricarboxylic acid (TCA) cycle, and inhibition of oxidative phosphorylation (OXPHOS) process (Li et al. 2023). However, M2 macrophages in the TME have a high glucose uptake capacity. Increased glucose uptake enhances the activity of the hexosamine biosynthetic pathway. It leads to the enhancement of O-GlcNAcylation on cathepsin B in TAMs, which in turn leads to increased secretion of cathepsin B in the TME and promotes tumor metastasis (Shi et al. 2022). In the B16 melanoma tumor model, STAT6 induces M2 macrophage polarization and inhibits TRIM24 expression in M2 macrophages, thereby inducing an immunosuppressive tumor niche (Yu et al. 2019). In addition, polarized M2 macrophages enhance phosphoglycerate kinase 1 (PGK1) threonine (T) 243 phosphorylation in tumor cells by secreting IL-6. Conversely, inhibition of this phosphorylation or neutralization of macrophage-derived IL-6 abrogates macrophage glycolysis, proliferation, and tumorigenesis (Zhang et al. 2018). A recent study also showed that macrophage polarization is closely related to the glycolytic process in Hepatocellular carcinoma. On the one hand, inhibiting the glycolysis process will reduce the ability of macrophages to express PD-L1, thereby restoring the immune function of T cells. On the other hand, inhibiting this process will also reduce the ability of TAMs to express anti-tumor IL-12p70 and promote tumor development (Lu et al. 2022a).

Hypoxia-inducible factor-1α (HIF-1α), as an important molecule in regulating macrophage function under hypoxic conditions, can induce the expression of related enzymes and transcription factors to promote glycolysis and the pentose phosphate pathway (PPP), thereby affecting the biological functions of macrophages. For example, pyruvate kinase M2, a key glycolytic enzyme derived from liver cancer cells, regulates the glycolysis process of macrophages in a HIF-1α-dependent manner (Lu et al. 2022a). At the same time, HIF-1α promotes the expression of pro-inflammatory genes in macrophages, enhances phagocytosis, affects the production of antimicrobial peptides and granzymes, and plays an important role in inflammatory responses (Li et al. 2023). In terms of PPP and TCA cycle, overexpression of glucose transporter 1 (GLUT1) in M1 macrophages has highly pro-inflammatory characteristics, leading to elevated glucose uptake and glucose metabolism by increasing the intermediates of PPP (Freemerman et al. 2014). Itaconate, an intermediate metabolite derived from the TCA cycle, regulates macrophage metabolism by inhibiting succinic acid oxidation. In addition, itaconate can exert an anti-inflammatory effect in activated macrophages (Lampropoulou et al. 2016). However, in the presence of NO, NO inhibits the activity of aconitase 2 and prevents carbon from entering the TCA cycle, thereby affecting the metabolic reprogramming of macrophages (Palmieri et al. 2020).

**Fig. 3:**
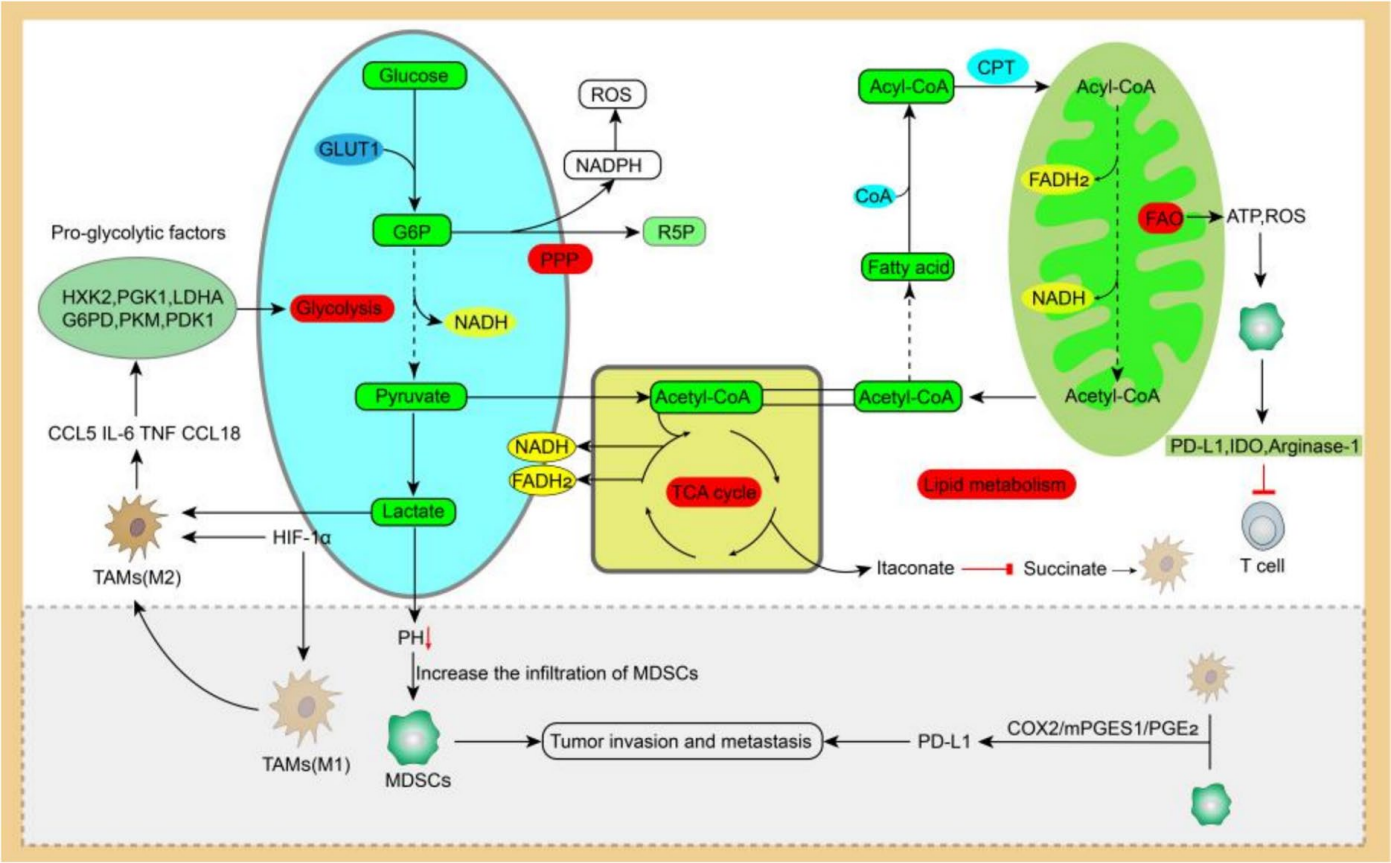
Effects of glucose metabolism and fat metabolism in the TME on TAMS and MDSCs. Lactate and HIF-1α induce M2 macrophages to secrete cytokines such as CCL5, IL-6, TNF, and CCL8, and promote the synthesis of pro-glycolytic factors (HXK2, PGK1, LDHA, G6PD, PKM, PDK1). HIF-1α also induces the polarization of M1 macrophages into M2 macrophages. Increased lactate leads to a decrease in the pH value in the TME, increases the infiltration of MDSCs and promotes tumor invasion and metastasis. The TCA cycle intermediate itaconate regulates macrophage metabolism by inhibiting succinate oxidation. FAO produces ATP and ROS and acts on MDSCs, while MDSCs inhibit the anti-tumor immunity of T cells by producing PD-1, IDO and Arginase-1. TAMs and MDSCs induce the expression of PD-L1 through COX2/mPGES1/PGE2, promoting tumor immune evasion

In MDSCs, The glycolytic pathway of MDSCs is regulated by HIF-1α, thereby enhancing the immunosuppressive activity of MDSCs (Corzo et al. 2010; Jian et al. 2017). In addition, the glycolysis product lactate can also stimulate the immunosuppressive property of MDSCs (Salminen et al. 2019). For example, increased lactic acid secretion by tumor cells leads to a decrease in the pH value in the TME, and the resulting acidic environment promotes tumor invasion and metastasis by increasing the infiltration of MDSCs (Fig. 3) (Husain et al. 2013). HIF-1α can activate glycolysis-related genes such as GLUT1, which is beneficial to the proliferation and immune suppression of MDSCs (Corzo et al. 2010) Overactivated glycolytic activity in TANs is associated with immunosuppressive and pro-tumor function of neutrophils (Wang et al. 2023). Neutrophils express PD-L1 under lactic acid induction, and neutrophils increase lactate concentration in TME by enhancing aerobic glycolysis in tumor cells, thus promoting N2 polarization of neutrophils (Deng et al. 2021a; Wang et al. 2021; Ye et al. 2022). In DCs, cancer cell-derived lactate attenuates the activation of human pDCs, leading to immune dysfunction and promoting tumor immune evasion (Raychaudhuri et al. 2019). Monocyte-derived human tolerogenic dendritic cells produce large amounts of lactic acid through glycolysis, and the resulting acidic environment conducive to immunosuppression inhibits the proliferation of T cells and induces the expansion of Tregs (Marin et al. 2019). During the oxidation stage of PPP, nicotinamide adenine dinucleotide phosphate (NADPH) oxidase (NOX) generates NADPH, and this process is the main source of ROS (Fig. 3) (Li et al. 2021d). When glucose and PPP-derived NADPH are limited, tumor-induced oxidative neutrophils can maintain ROS production and inhibit T cell function, promoting tumor growth (Rice et al. 2018). PMN-MDSCs can also exhibit higher NOX activity, thereby producing more ROS to inhibit T cells (Li et al. 2021d).

### *Lipid metabolisim*

In addition to serving as membrane structures, lipids also provide energy sources for cells when nutrients are limited (Fig. 3) (Efeyan et al. 2015). For example, MDSCs can absorb fatty acids in the TME (Yan et al. 2019), and tumor-infiltrating MDSCs increase fatty acid oxidation (FAO). Inhibiting FAO can block the immunosuppressive function of tumor-infiltrating MDSCs, allowing T cells to exert anti-tumor effects (Hossain et al. 2015). Furthermore, fatty acid transporter protein controls the inhibitory activity of PMN-MDSCs by increasing PGE2 synthesis and arachidonic acid uptake (Veglia et al. 2019). However, the increased metabolism of arachidonic acid is related to the tumor-promoting function of TAMs (Zheng et al. 2020).

Fatty acid metabolism in TAMs can confer pro-tumor and anti-tumor phenotypes on TAMs. And high levels of fatty acid oxidation and proliferation were observed in TAMs (Su et al. 2020). Studies have shown that TAMs enhance the proliferation of T cells in the TME by producing large amounts of extracellular vesicles (EVs). TAM-EVs are rich in lipid metabolism proteins such as COX-1 and some cytochrome P450 enzymes, and TAM-EVs are associated with good prognosis in cancer patients (Cianciaruso et al. 2019), this study supports the anti-tumor function of fatty acid metabolism in TAMs. However, more studies have demonstrated its tumor-promoting functions. The abundant endoplasmic reticulum and free cholesterol in macrophages can promote the esterification of cholesterol acetyl-CoA acetyltransferase 1, which in turn leads to the production of more free cholesterol and increases fat-induced inflammatory signals, such as TLRs and NF-κB, this signaling pathway leads to changes in macrophage lipid metabolism (Li et al. 2023). In addition, lipid metabolism can promote the activation and polarization of TAMs. Peroxisome proliferator-activated receptor gamma (PPAR-γ)-dependent FAO upregulation mediates M2 polarization of TAMs. This mechanism is due to S100A4 controlling the upregulation of PPAR-γ, PPAR-γ is the transcription factor required for FAO induction during the tumor-promoting progression of TAMs (Liu et al. 2021). And caspase-1 can inhibit FAO by cleaving PPAR-γ, helping TAMs differentiate and promote tumor progression (Niu et al. 2017). Because PPARγ, which affects M2 polarization, mainly promotes fatty acid oxidation and mitochondrial production at the transcriptional level, and couples with PGC-1β to regulate the production of M2 macrophage marker Arg1. Therefore, knocking down PPARγ in macrophages in vivo and in vitro inhibits M2 macrophage activation and reduces Arg1 production (Caputa et al. 2019). In addition, Treg cells maintained the metabolic adaptability, mitochondrial integrity, and survival rate of M2-like TAMs by blocking the activation of sterol regulatory element binding protein 1 (SREBP1)-mediated fatty acid synthesis and inhibiting the CD8 + T cell-IFN-γ axis (Liu et al. 2019). These studies suggest that tumor cells may affect TAMs polarization by regulating cholesterol metabolism, thereby promoting tumor development. Due to the important role of lipid metabolism in tumor development, especially the crosstalk between lipid metabolism and TAMs, antitumor drugs targeting lipid metabolism, such as PPAR agonists/antagonists, fatty acid binding protein inhibitors, and fatty acid synthase inhibitors, are currently under development. These drugs affect tumor growth and metastasis by regulating lipid metabolism, affecting tumor-associated immune cells, and inhibiting the function of lipid transport proteins (Ren et al. 2024).

Neutrophils have been shown to be regulated by cholesterol levels and are also able to recognize certain effector substances in the microenvironment through fatty acid transporters to regulate their role in inflammation, and FAO can be performed to compensate for the reduced ROS levels caused by the lack of glucose in the TME (Aguilar-Ballester et al. 2020; Bodac and Meylan 2021; Jiang et al. 2022). For example, mesenchymal cells resident in the lung can induce neutrophils to accumulate lipids before metastasis. When breast cancer cells metastasize and colonize in the lungs, they acquire higher growth capacity and survival levels by absorbing lipids previously accumulated by lung neutrophils, proving that infiltrating neutrophils can act as an energy source for lung metastasis of breast cancer cells (Li et al. 2020). However, The accumulation of lipids in DCs will destroy their function (Peng et al. 2021). Studies have shown that in mesothelioma, factors secreted by tumor cells promote the accumulation of lipids in DCs, thereby reducing their ability to present antigens (Gardner et al. 2015). This mechanism is also reflected in ovarian cancer. The intrinsic fatty acid synthase in ovarian cancer cells leads to abnormal accumulation of lipids in tumor-infiltrating DCs, affects the anti-tumor activity of T cells, and promotes tumor progression (Jiang et al. 2018).

### Amino acid metabolism

In the TME, tumor cells rely on supply levels of amino acids to maintain their rapid proliferation. By driving the synthesis of nucleotides, amino acids can promote the development, metastasis and immune evasion of tumor cells, and can also maintain the redox homeostasis of cells (Li and Zhang 2016; Vettore et al. 2020; Wang and Wan 2022). TAMs exhibit high aryl hydrocarbon receptor (AhR) activity, and studies have shown that tryptophan-derived microbial metabolites can activate AhR in TAMs, thereby suppressing anti-tumor immunity. Deletion or inhibition of AhR reduced the growth of pancreatic ductal adenocarcinoma and improved the efficacy of ICB (Hezaveh et al. 2022). Furthermore, ornithine decarboxylase in macrophages exacerbates colitis and promotes colitis-associated colon carcinogenesis by impairing M1-type immune responses (Singh et al. 2018). Arginine is a semi-essential amino acid that is metabolized into ornithine and urea by cytoplasmic arginase 1 (ARG1), mitochondrial arginase 2 (ARG2) or nitric oxide synthase. ARG2 drives neuroblastoma cell proliferation by regulating arginine metabolism. And arginine metabolism polarizes infiltrating monocytes to an M1 macrophage phenotype and releases IL-1β and TNF-α in a RAC-α serine/threonine protein kinase-dependent manner (Fultang et al. 2019). In addition, arginine induces macrophage activation by promoting NO synthesis (Wei et al. 2020). And activated TAMs and MDSCs can also inhibit the immune function of T cells by expressing Arg1 (Rodriguez et al. 2017). Among them, MDSCs mainly inhibit the proliferation of T cells by consuming arginine in the TME (Gabrilovich and Nagaraj 2009; Halaby and McGaha 2021). There are also studies showing that MDSCs cause impairment of the immunosuppressive function of T cells through NO-related pathways (Raber et al. 2014). TANs can also secrete Arg1 to cause the degradation of arginine, thus inhibiting anti-tumor responses (Jiang et al. 2022). In DCs, enzymes involved in arginine metabolism such as Arg1 are related to the function of DCs (Rodriguez et al. 2017). For example, in a mouse model of epithelial ovarian cancer, extracellular vesicles containing Arg1 are transported into lymph nodes and taken up by DCs, thereby inhibiting the proliferation of T cells and further leading to tumor growth and immune evasion (Czystowska-Kuzmicz et al. 2019). Metabolism of tryptophan is also involved in the metabolic programming and function of myeloid cells (Halaby and McGaha 2021; Wei et al. 2020). Tryptophan, as an essential amino acid for the human body, is the substrate of indoleamine-2,3-dioxygenase 1 (IDO1), which is mainly decomposed through the kynurenine pathway to produce the immunosuppressant kynurenine and other physiologically active substances. For example, overexpression of IDO1 in breast cancer causes the accumulation of kynurenine and the consumption of tryptophan in tumor cells, inhibiting T cell-mediated immune responses (Heng et al. 2016). In addition, MDSCs promote the release of immunosuppressive factors and inhibit the proliferation of T cells under the induction of IDO (Yu et al. 2013). Although there has been significant progress in the study of amino acid metabolism of myeloid cells in tumor cells, there are very few studies on the amino acid metabolism of eosinophils and basophils in tumor tissues. In addition, due to the diversity of amino acid types, the metabolism of other types of amino acids in the TME should also receive extensive attention.

## Summary and outlook

Over the past few decades, the importance of bone marrow-derived cells in the TME has been widely realized, and more and more studies demonstrated the role of myeloid cells in tumor growth and development, invasion and migration, as well as metabolism and treatment. Myeloid cells are components of innate immune system, participating in host defense against pathogens and regulating innate and adaptive immune responses (Güç and Pollard 2021; Wang et al. 2022b). Wang, Y et al. summarized the strategies for immunotherapy by targeting myeloid cells and the current clinical trials of myeloid cell therapy (Wang et al. 2022b), including enhancing the differentiation and proliferation of myeloid cells to change the composition of cells in the TME, or inhibiting their functions, regulating the metabolic reprogramming of bone marrow cells, etc.

We comprehensively summarized the role of myeloid cells (including macrophages, neutrophils, myeloid-derived suppressor cells, dendritic cells, basophils, and eosinophils) in regulating tumor immunity in the tumor microenvironment, the impact on cancer invasion and metastasis and interactions with glucose metabolism, lipid metabolism, and amino acid metabolism are discussed, and the complexity of cancer treatment is discussed, as well as their contributions leading to the complexity of cancer treatment. In future research work, the interactions between myeloid cells and other cells in the TME should be further explored, and consider the impact of different tumor types, different locations of tumor occurrence, and the host's normal physiological activities such as diet, exercise, lifestyle habits, or other types diseases of host on the TME. In addition, with the continuous development of single-cell omics and other cutting-edge technologies with higher resolution such as spatiotemporal omics, it is promising that the exact interactive network of distinct cell populations in TME will be dissected in near future, thereby leads to further development of better therapeutic strategies for treating cancer.

## Data Availability

No datasets were generated or analyzed during the current study.

## Abbreviations

AhR: Aryl hydrocarbon receptor
ANGPT1: Angiopoietin 1
ANGPT2: Angiopoietin 2
APC: Antigen presenting cell
ARG1: Arginase 1
Arg-1: Arginase-1
ARG2: Arginase 2
cDC1: Classical dendritic cells type 1
cDC2: Classical dendritic cells type 2
CPEB3: Cytoplasmic polyadenylation element binding protein 3
CTLs: Cytotoxic T lymphocytes
DCs: Dendritic cells
EGF: Epidermal growth factor
EMT: Epithelial-to-mesenchymal transition
EPO: Eosinophilic peroxidase
EVs: Extracellular vesicles
FAO: Fatty acid oxidation
G-CSF: Granulocyte colony-stimulating factor
GLUT1: Glucose transporter 1
GM-CSF: Granulocyte macrophage colony-stimulating factor
G-MDSC: Granulocytic MDSC
HGF: Hepatocyte growth factor
HIF-1α: Hypoxia-inducible factor-1α
HMGB1: High motility group box protein 1
HSCs: Hematopoietic stem cells
ICB: Immune checkpoint blockade
ICC: Intrahepatic cholangiocarcinoma
IDO1: Indoleamine-2,3-dioxygenase 1
IFN-γ: Interferon-gamma
LPS: Lipopolysaccharides
M-CSF: Macrophage colony-stimulating factor
MDSC: Myeloid suppressor cells
MHC II: Major Histocompatibility Complex Class II
M-MDSC: Mononuclear MDSC
MMP: Matrix metalloprotein
NADPH: Nicotinamide adenine dinucleotide phosphate
NETs: Neutrophil extracellular traps
NK: Natural killer
NO: Nitric oxide
NOX: NADPH oxidase
OXPHOS: Oxidative phosphorylation
PDCs: Plasmacytoid dendritic cells
PDGF: Platelet-derived growth factor
PGE2: Prostaglandin E2
PGK1: Phosphoglycerate kinase 1
PMN: Premetastatic niche
PMN-MDSC: Polymorphonuclear MDSC
PPAR-γ: Peroxisome proliferator-activated receptor gamma
PPP: Pentose phosphate pathway
RIG-I: Retinoic acid-inducible gene I
ROS: Reactive oxygen species
SREBP1: Sterol regulatory element binding protein 1
TAMs: Tumor-associated macrophages
TANs: Tumor-associated neutrophils
TCA: Tricarboxylic acid
TGF-β: Transforming growth factor-beta
TLRs: Toll-like receptors
TME: Tumor microenvironment
TNF-α: Tumor necrosis factor-alpha
VEGF: Vascular endothelial growth factor
